# Optic disc cupping characteristics of normal pressure hydrocephalus patients with normal-tension glaucoma

**DOI:** 10.1038/s41598-019-39526-2

**Published:** 2019-02-28

**Authors:** Nozomi Igarashi, Megumi Honjo, Shotaro Asano, Kiyoshi Takagi, Makoto Aihara

**Affiliations:** 1 Department of Ophthalmology, Graduate School of Medicine, The University of Tokyo, Tokyo, Japan; 2 Department of Normal Tension Hydrocephalus, Kashiwa Tanaka Hospital, Chiba, Japan

## Abstract

We examined the potential association of idiopathic normal pressure hydrocephalus (iNPH) with the generation of normal-tension glaucoma (NTG), to explore possible relationships between intracranial pressure (ICP) and the presence of glaucoma, and to compare disc morphology of NTG patients with or without iNPH. We investigated 20 iNPH patients, examined the prevalence of glaucoma, and compared the optic discs of NTG patients with iNPH (n = 11) and age-matched NTG patients without iNPH (n = 16). All data were collected prior to the treatment of iNPH, to eliminate the possibility that the treatment may have contributed to the progression of NTG. The diagnoses of NTG were made using visual field data, intraocular pressure measurements, fundoscopy, and optical coherence tomography (OCT). Using OCT, the optic nerve disc depth was also measured. The ICP was higher in the iNPH with NTG compared to iNPH without NTG (p = 0.0425), and the cupping depths of the discs of NTG patients with iNPH were significantly shallower compared with those of NTG patients without iNPH (p = 0.0097). Based on the difference in cupping depth, NTG patients with iNPH may have a different morphology from typical glaucoma patients, which could in turn reflect a different pathogenesis compared to NTG patients without iNPH.

## Introduction

Glaucoma is the second leading cause of blindness globally, and is characterized by abnormal increases in intraocular pressure (IOP) that can damage the optic nerve^1–3^. Although elevated IOP is one of the most important risk factors for primary open-angle glaucoma (POAG), which is the most common subtype of glaucoma, many POAG patients have normal IOP measurements, a condition known as normal-tension glaucoma (NTG)^4^. Reducing the IOP is the only effective therapy to prevent visual impairment and blindness, not only in hypertensive, but also in normotensive individuals; it has been suggested that factors other than the IOP, such as hemodynamic abnormalities, vasospasm, systemic hypotension, and autoimmune diseases may be more important in NTG patients^5–8^. In addition to these vascular risk factors, it has been suggested that differences in CSF pressure may influence the propensity toward glaucomatous damage. Several studies reported that intracranial pressure (ICP) was lower in patients with NTG compared to those with POAG and non-glaucomatous control subjects, suggesting that low ICP may result in a high translaminar pressure gradient (TPG) and impairment of axonal transport in the lamina cribrosa^9,10^. Moreover, it has been reported that a low ICP contributes to worsening NTG^11,12^; low ICP may lead to an abnormally high TPG in normal IOP, and could result in optic nerve damage at the lamina cribrosa^11,12^. However, this cannot be easily proven, because it is difficult to measure the actual intraorbital CSF pressure in the subarachnoid space of the optic nerve. In addition, it was recently reported that the ICP was not low in NTG patients^13^, so whether low ICP is clinically related to the occurrence of glaucoma is still controversial. In addition, the results of a couple of experimental studies on the mechanisms of ICP and NTG failed to support this theory^14^, indicating that a low ICP did not result in bowing of the lamina cribrosa or optic disc cupping^14,15^. It has also been theorized that a decreased ICP can be the consequence of reduced CSF production, and may reflect reduced CSF turnover, with CSF circulatory dysfunction resulting in reduced clearance of toxic substances from the subarachnoid space surrounding the optic nerve^15^.

As mentioned above, the precise relationship between the condition of the CSF and glaucomatous damage is still unclear. We therefore focused on idiopathic normal pressure hydrocephalus (iNPH), which is a disease characterized by reduced CSF turnover. iNPH is diagnosed when patients present with a symptomatic triad of cognitive dysfunction, gait disturbances, and urinary incontinence, in spite of a normal ICP. Notably, iNPH has been reported to have a higher prevalence rate in glaucoma patients^12,16,17^. It has been suggested that the circulatory physiology of the CSF is significantly accentuated in iNPH patients, while the ICP usually remains within the normal range^18–21^. Several previous studies have reported a possible association between glaucoma and iNPH; for example, Chang *et al*. reported that the prevalence of glaucomatous disease in patients with iNPH was 18.1%, which was much higher than that of age-matched controls with hydrocephalus (5.6%)^16,22^. However, to the best of our knowledge, no study has reported the relationship between ICP and the prevalence of glaucoma in iNPH patients. Moreover, no reports have assessed the characteristics of iNPH patients with glaucoma. The objectives of the present study were therefore to confirm the prevalence of glaucoma in iNPH patients, explore possible associations between ICP and the presence of glaucoma, and investigate disc morphology to determine whether the pathological features of glaucoma patients with iNPH showed any differences, in terms of optic disc morphology and functional test results, compared to those of NTG patients without iNPH.

## Results

### Demographic data of the study population

Table 1 lists the demographic data of the study population. Of the 20 iNPH patients included in the study, 11 (55%) were classified as iNPH+/NTG+.

**Table 1:** Demographic data of the study population.

Variables	iNPH(+)		iNPH(−)	p value
	NTG(−)	NTG(+)	NTG	
**Patients (n)**	9	11	16	
**Number of eyes (n)**	18	22	27	
**Gender (male:female)**	6:3	6:5	7:9	NS*
**Age (years)**				
Mean ± SD	77.2 ± 5.70	77.63 ± 3.90	73.75 ± 2.65	NS**
[range]	63–85	67–87	60–83	
**IOP (mmHg)**				
Mean ± SD	11.7 ± 2.5	12.6 ± 2.7	13.7 ± 2.2	^†^<0.005**
[range]	9–16	9–18	10–19	
**Disc depth (μM)**				
Mean ± SD	221.8 ± 73.7	228.1 ± 75.1	333.3 ± 113.2	^††^<0.0001**
[range]	86–351	115–368	217–616	
**Vertical C/D ratio**				
Mean ± SD	0.4 ± 0.07	0.55 ± 0.11	0.55 ± 0.08	NS**
[range]	0.3–0.5	0.4–0.8	0.4–0.7	
**Glaucomatous optic disc appearances (FI:GE:MY:SS)**	—	6:16:0:0	5:15:2:2	
**MD**				
Mean ± SD	—	−5.309545 ± 2.446755	−4.131154 ± 1.870012	NS**
[range]	—	−25.87~−0.22	−18.07~2.11	

*Fisher’s exact test; **Analysis of variance.

^†^Statistically significant difference between the non-NTG-iNPH and non-iNPH-NTG groups (Steel-Dwass test).

^††^Statistically significant difference between the iNPH-NTG and non-iNPH-NTG groups (Steel-Dwass test). IOP, intraocular pressure; FI, focal ischemic; GE, general enlargement; MY, myopic glaucomatous; SS, senile sclerosis; MD, mean deviation; NTG, normal-tension glaucoma; iNPH, idiopathic normal pressure hydrocephalus.

The mean IOP was 11.7 ± 2.5, 12.6 ± 2.7, and 13.7 ± 2.2 mmHg for the iNPH+/NTG-, iNPH+/NTG+, and iNPH−/NTG+ groups, respectively (Table 1). The IOP was significantly lower in the iNPH+/NTG- group compared to the iNPH−/NTG+ group, but there was no significant difference in IOP between the iNPH+/NTG+ and iNPH−/NTG+ groups (Table 1).

**Figure 1:**
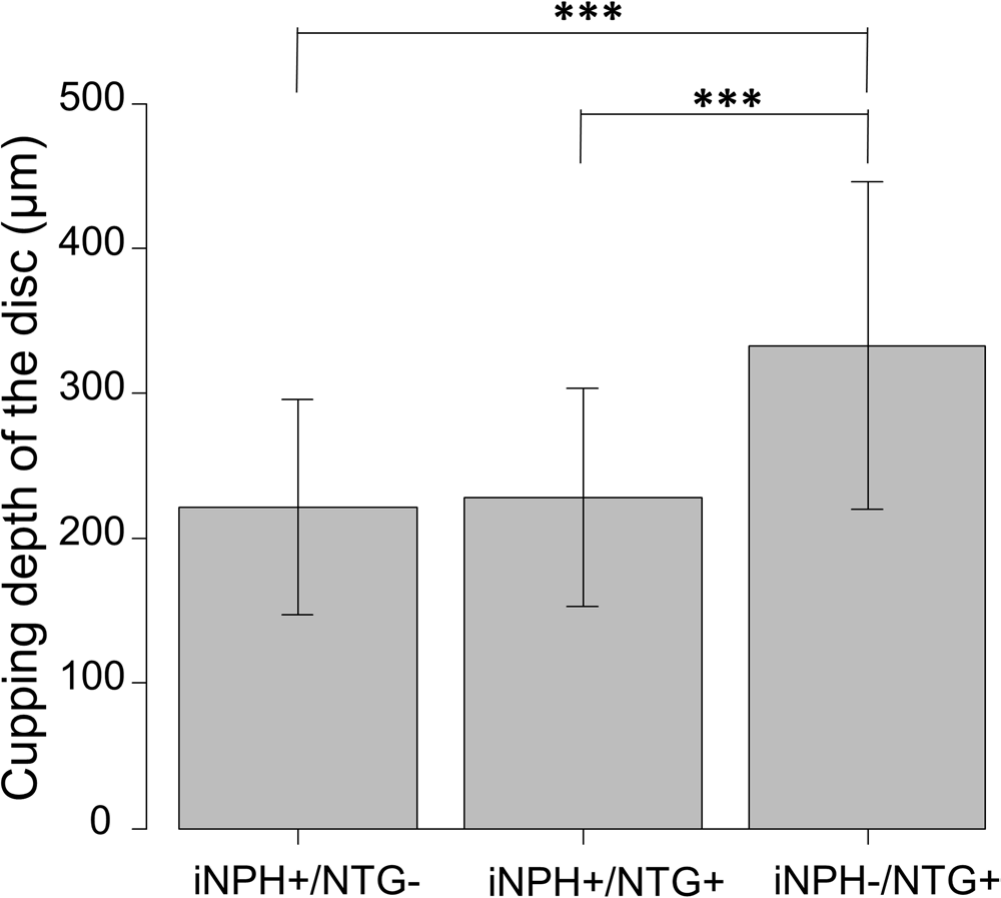
Comparison of the cupping depth of the optic disc among the iNPH+/NTG-, iNPH+/NTG+, and iNPH−/NTG+ groups. The cupping depth of the optic disc was measured using SD-OCT for patients in the iNPH+/NTG-, iNPH+/NTG+, iNPH−/NTG+ groups; the cupping depth for the iNPH−/NTG+ group was significantly greater than that for the other groups (p < 0.001). ^***^P < 0.001.

The mean cupping depth of the discs was 221.8 ± 73.7, 228.1 ± 75.1, and 333.3 ± 113.2 μm for the iNPH+/NTG-, iNPH+/NTG+, and iNPH−/NTG+ groups, respectively (Table 1). The cupping depth of the discs was significantly greater in the iNPH−/NTG+ group (p < 0.0001) (Table 1; Fig. 1).

### Comparison between the iNPH+/NTG+ and iNPH+/NTG- groups

**Figure 2:**
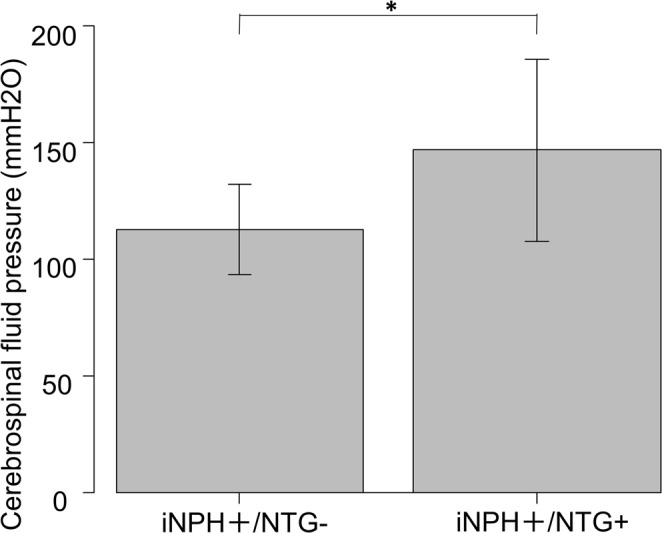
Comparison of cerebrospinal pressure between the idiopathic normal pressure hydrocephalus-positive/normal-tension glaucoma-negative (iNPH+/NTG-) and iNPH+/NTG+ groups. The cerebrospinal fluid (CSF) pressure between the iNPH+/NTG- and iNPH+/NTG+ groups was measured using the lumbar puncture method, and the CSF was found to be significantly higher in the iNPH+/NTG+ group (p < 0.05). ^*^P < 0.05.

Factors associated with the presence of NTG in iNPH patients were compared between the iNPH+/NTG- and iNPH+/NTG+ groups (Table 2). Although MMSE, gait and cognitive function showed no significant difference between the two groups, a higher ICP was significantly correlated with the prevalence of NTG in iNPH patients (p = 0.0425, *t*-test; Table 2; Fig. 2).

**Table 2:** Comparison between the iNPH+/NTG+ and iNPH+/NTG− groups.

Variables	non-NTG-iNPH	NTG-iNPH	p value^‡^
**ICP (mmHg)**			
Mean ± SD	8.3 ± 1.4	10.8 ± 2.9	^†^<0.005***
[range]	5.9–9.9	5.7–14.1	
**MMSE prior to CSF tapping**			
Mean ± SD	22.33 ± 5.29	23.27 ± 4.73	NS***
[range]	12–30	16–28	
**MMSE after CSF tapping**			
Mean ± SD	24.22 ± 5.22	25.09 ± 4.09	NS***
[range]	18–30	16–30	
**Cognitive function (improved: no improvement) (n)**	6:3	6:5	NS***
**Gait disturbance (improved: no improvement) (n)**	8:1	6:5	NS***
**P-tau**			NS***
Mean ± SD	40.9 ± 12.7	51.8 ± 25.3	
[range]	27.8–63.2	32.3–109.0	

^‡^Two groups compared using the *t*-test.

^†^Statistically significant difference between the non-NTG-iNPH and NTG-iNPH groups (Steel-Dwass test).

SD, standard deviation; ICP, intra cranial pressure; MMSE, Mini-Mental State Examination; CSF, cerebrospinal fluid; p-tau, tau protein; NTG, normal-tension glaucoma: iNPH, idiopathic normal tension hydrocephalus.

P-tau is a possible biomarker for iNPH. The mean P-tau level in the CSF was 40.9 ± 12.7 and 51.8 ± 25.3 pg/mL for the iNPH+/NTG- and iNPH+/NTG+ groups, respectively. The iNPH+/NTG+ group showed a relatively higher P-tau level, but showed no significant difference between iNPH+/NTG+ and iNPH+/NTG- groups (p = 0.358), and no significant correlation was seen between the CSF p-tau concentration and presence of NTG.

### Comparison between the iNPH+/NTG+ and iNPH−/NTG+ groups

As shown in Fig. 1, the cupping depth of the discs was significantly greater for the iNPH−/NTG+ group compared to iNPH+/NTG- group (p < 0.001), although there was no significant difference in the vertical C/D ratio or mean deviation (MD) between these two groups (Table 1). In the iNPH +/NTG+ group, 17 eyes (77%) had glaucomatous visual field defects, and 5 (23%) were preperimetric, while 20 eyes (74%) had glaucomatous visual field defects, and 7 (26%) were preperimetric in iNPH−/NTG+ group.

**Figure 3:**
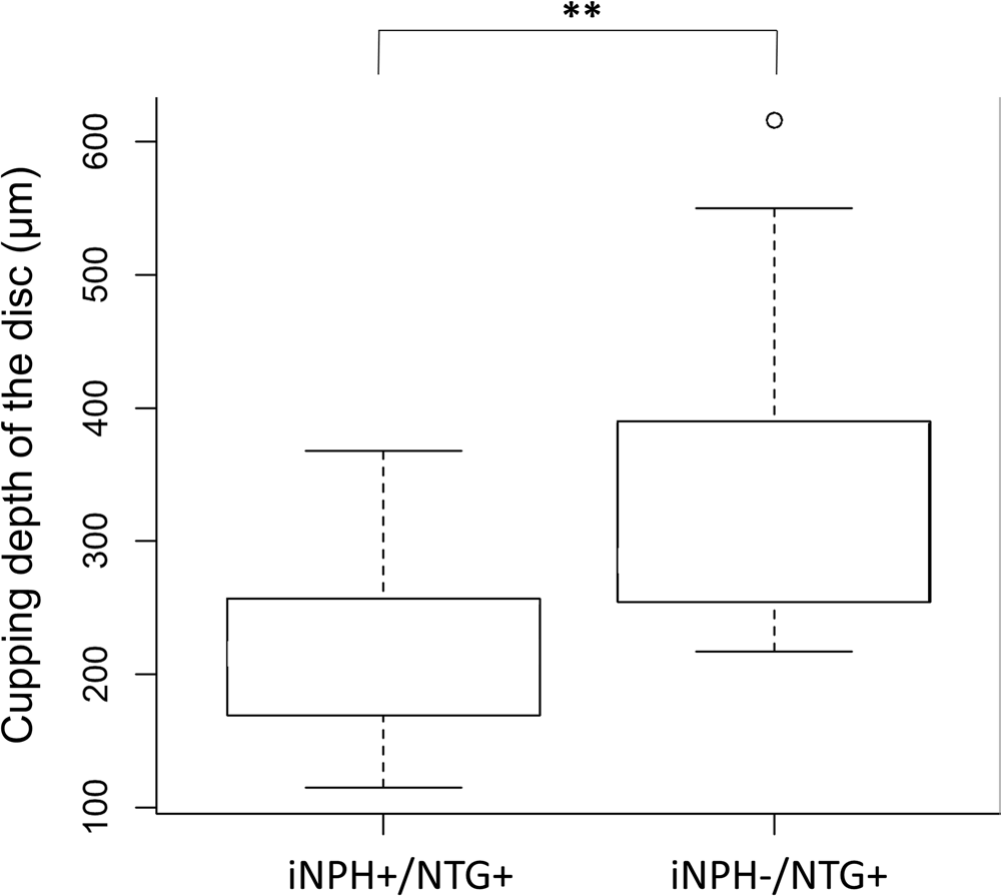
Linear combination model analysis of the cupping depth of the optic disc: comparison between the iNPH+/NTG+ and iNPH−/NTG+ groups. The cupping depth of the optic disc was measured by SD-OCT for the iNPH+/NTG+ and iNPH−/NTG+ groups using a linear combination model; that of the iNPH−/NTG+ group was significantly greater compared to the iNPH+/NTG+ group (p < 0.01). ^**^P < 0.01.

**Figure 4:**
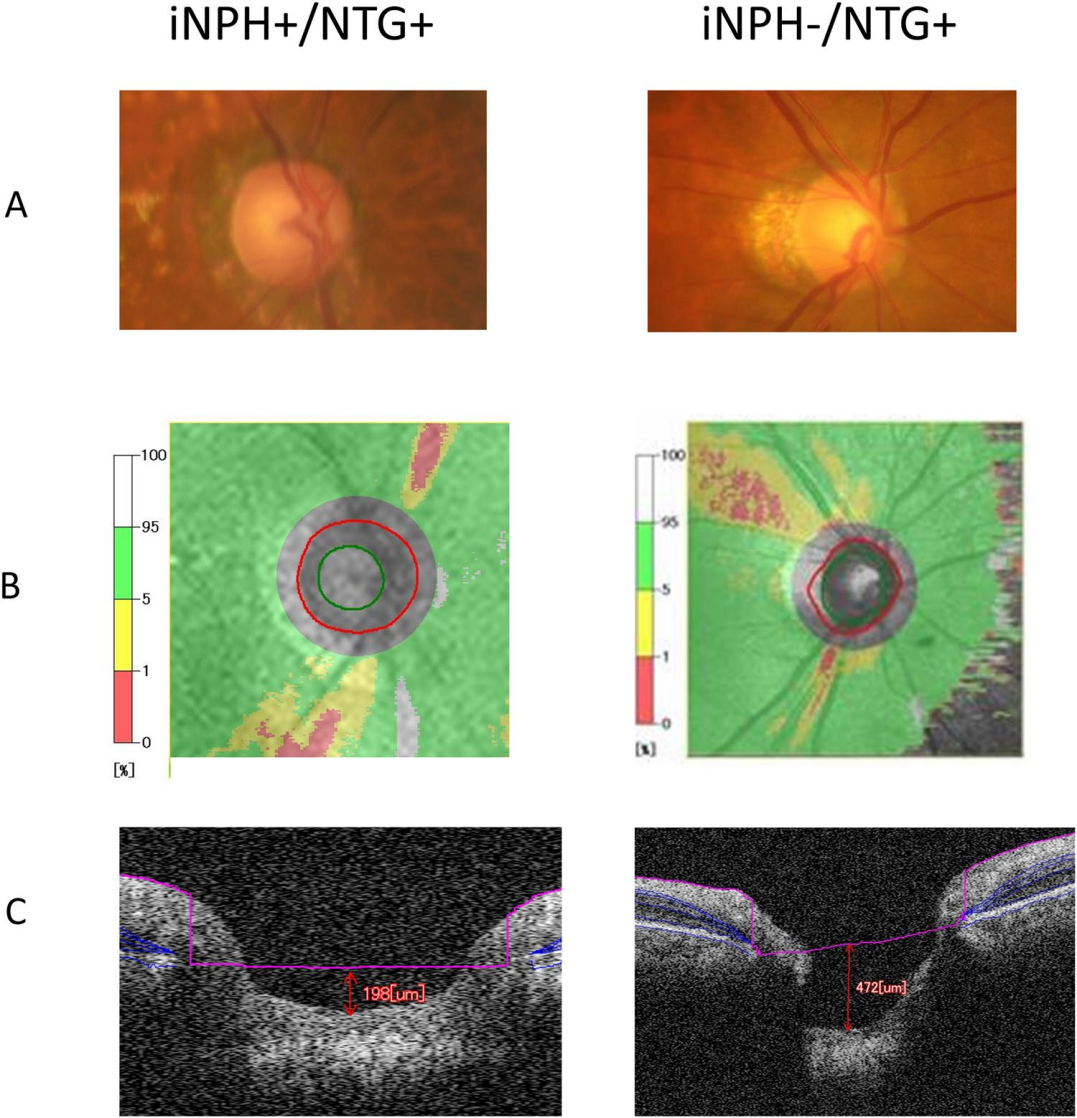
Representative disc photo, visual field and spectral domain-optical coherence tomography (SD-OCT) for iNPH+/NTG+ and iNPH−/NTG+ patient. Example measurement of the disc photo (**A**), disc OCT (**B**), and cupping depth of the optic disc (**C**) are shown. In (**B**) the green circle shows the inner circumference (excavating part) of the disc, and the red circle shows the outer circumference of the disc. On the left, 83-year-old, iNPH+/NTG+ patient (Mean deviation, −3.59 dB), and on the right, 73-year-old, iNPH−/NTG+ (Mean deviation, −5.45 dB) patient’s data is shown. On the acquired B-scan image, Bruch’s membrane was first marked, and the Bruch’s membrane openings were connected to form a reference plane (pink line on the image). The distance from the reference plane to the bottom of the optic disc was taken as the cupping depth of the disc.

We further used a linear combination model to ascertain the difference in cupping depth of the discs between the NTG patients with and without iNPH, because our analyses included both eyes of some patients in the study. The linear combination model analysis comparing the iNPH+/NTG+ and iNPH−/NTG+ groups revealed that the cupping depth of the discs was significantly greater in the iNPH−/NTG+ group (p = 0.0097; Figs 3 and 4), although there was no significant difference in the mean MD between the two groups.

## Discussion

In the present study, we determined the frequency of comorbid NTG and iNPH, investigated possible relationships between the ICP and the presence of glaucoma, and compared the glaucomatous disc morphology of NTG patients between those with and without iNPH. The major findings were as follows: (1) the rate of NTG was significantly higher in iNPH patients, (2) the ICP was not lower, but rather higher, in NTG patients with iNPH compared to non-NTG iNPH patients, and (3) NTG patients with iNPH showed characteristically shallow optic disc cupping compared to NTG patients without iNPH.

Many studies have suggested that glaucoma shares common features with other well-known age-related neurodegenerative diseases, such as Alzheimer’s disease (AD), Parkinson’s disease, and iNPH, and it has been reported that the incidence rate of glaucoma among patients with these neurodegenerative diseases is higher than that of patients without them^23–25^. In a Japanese study, Tamura *et al*. noted open-angle glaucoma (OAG) in 23.8% of 172 patients with AD, which was significantly higher than the rate of 9.9% for 176 control subjects^24^. In the Tajimi Study^26^, the estimated prevalence of glaucoma for Japanese persons aged >40 years was 5.0%. It should also be noted that approximately 90% of OAG patients in Japan suffer from NTG^24^. It has been determined that AD is one of the risk factors for glaucoma, especially in NTG patients^27^. Regarding iNPH patients, there have been a few previous reports regarding the relationship between iNPH and glaucoma^15,21^. Chen *et al*. reported a case of worsening NTG immediately following ventriculoperitoneal (VP) shunt placement in iNPH patients^11^. In a case-control study, Chang *et al*. reported a higher prevalence of glaucomatous disease, of 18.1%, among 72 patients with iNPH, which was much higher than that of age-matched iNPH (-) controls with hydrocephalus (5.6%)^15,21^. Recently, a high prevalence of NTG (40.9%) was reported among 22 iNPH patients who underwent VP shunt placement^12^. In the present study population, the prevalence of presumed NTG in iNPH patients was significantly higher than the general estimate (55%; Table 1); nevertheless, our results were generally consistent with previous studies, especially that of Gallina *et al*.^12,15^. Overall, this is the first report revealing the high frequency of NTG comorbid with untreated iNPH. Further multicenter studies will be needed to determine the actual prevalence of NTG in iNPH patients, and to determine the characteristics of neurodegeneration of the optic nerve, as iNPH is a rare disease with a reported prevalence of only approximately 0.5% in the general population >65 years of age^28^. However, the higher incidence of glaucoma among patients with these neurodegenerative diseases implies that neurodegenerative disease-related optic nerve degeneration, which could be refractory to treatment for IOP reduction, should be recognized and considered along with IOP-dependent optic nerve degeneration^29,30^.

Regarding a possible causal link between the high incidence rate of NTG in neurodegenerative disease and the pathogenesis of glaucoma, two main factors have been proposed: low ICP and decreased CSF turnover^14^. Concerning lower ICP, recent studies reported that the ICP was lower in NTG patients compared to POAG and non-glaucomatous control subjects^9,10^, suggesting a potential pathological role for an abnormally low ICP in the development of OAG, especially in NTG patients^5–8^. The most commonly proposed mechanisms linking low ICP to the progression of NTG are high TPG, the difference between IOP and intraorbital CSF pressure in the subarachnoid space of the optic nerve, and the resulting impairment of axonal transport in the lamina cribrosa^9^. Berdahl *et al*. reported that the mean ICP, as measured by lumbar puncture, was 33% lower in a group of 28 patients with POAG than in a control group of 49 non-glaucomatous patients^31^. Another prospective study confirmed this result, reporting that the ICP was significantly lower in an NTG group (9.5 ± 2.2 mmHg; p = 0.013) than in a high-tension glaucoma group (11.7 ± 2.7 mmHg), or a control group (12.9 ± 1.9 mmHg)^10^. In the same study, the extent of glaucomatous VF loss was positively correlated with the IOP, and negatively correlated with CSF pressure, suggesting that patients whose CSF pressure is lower than the average with normal IOP could tend to show progressive glaucomatous damage. Although it has been previously shown that the CSF pressure measured by lumbar puncture well correlated with ICP^32^, as far as we know, it has not been clarified whether the CSF pressure is the same as the intraorbital CSF pressure so far, since there is no way to measure the actual intraorbital pressure in living humans. Thus, the actual relationship between ICP and pressure on the optic disc, including trans-lamina cribrosa pressure, is currently not definitively known.

In the present study, we compared the ICP between iNPH+/NTG- and iNPH+/NTG+ groups, with the expectation that a lower ICP may be related to the presence of glaucoma in iNPH patients. However, contrary to this expectation, the mean ICPs of the iNPH+/NTG- and iNPH+/NTG+ patients were 8.3 ± 1.4 mmHg (112.9 ± 19.4 mmH_2_O) and 10.8 ± 2.9 mmHg (146.8 ± 39.1 mmH_2_O), respectively. The ICP of NTG patients was not lower, and in fact was higher, compared to that of patients not diagnosed with NTG, although all obtained data were within the normal ICP range.

Based on these results, we hypothesize that decreased CSF turnover or fluctuation of the ICP may account for the pathogenesis of NTG in iNPH patients, as has been suggested previously^16^. iNPH is a disorder characterized by decreased CSF absorption, where it is assumed that the ICP may generally increase during the initial stage of the disease, thereby leading to ventricular enlargement followed by a return of the ICP to a normal level as the ventricles are enlarged^19^. Even if the measured ICP remains within the normal range at the site of lumbar puncture, it has been suggested that the ICP fluctuates intermittently with slow and rhythmic oscillations^33^. From a mechanical perspective, it has been shown that a pulsatile mechanical load has a more dramatic effect on cell physiology than a steady mechanical pressure or stress^34^. Because IOP fluctuation is known as an independent risk factor for ganglion cell damage^35^, it seems reasonable to speculate that the fluctuation of trans-lamina cribrosa pressure may play an important role in the pathogenesis of NTG in iNPH patients.

In addition, iNPH is also characterized by reduced CSF turnover, where it is decreased to two-thirds of the normal level^36^. Lindén *et al*. recently reported that ICP levels and CSF turnover had no effect on the occurrence of NTG, whereas impaired flow of fluid between the CSF space and optic nerve subarachnoid space might be an important trigger of NTG^13^. Impaired flow may cause accumulation of neurotoxins or diminished nutrition for axons or neurons. In iNPH, it has been reported that decreased turnover of the CSF resulted in a decrease in the clearance of β-amyloid and tau, which are known as major biomarkers for AD and iNPH^37^. Other neurotoxic substances, such as reactive oxygen species, are known to be produced continuously by cells, and the turnover of such agents are crucial in protecting retinal ganglion cell (RGC) axons at the optic nerve, where such agents can cause optic nerve damage. Malnutrition of the optic nerve has been suggested to cause impaired axonal transportation, leading to RGC death^38^. Decreased CSF turnover, and the resulting accumulation of neurotoxic substances, could at least in part be one of the pathological events involved in the development of NTG in iNPH patients. This implies that during pathogenesis of NTG in iNPH patients, optic nerve axons may be impaired around the subarachinoid space, subsequently resulting in disturbances in axonal flow or the reaction of supporting glial cells, finally resulting in structural changes followed by a pattern of optic nerve degeneration that may differ from the typical pressure-dependent axon damage induced by lamina deformation. Our data indicating a shallower cupping depth of the disc tend to support the mechanism of axon loss in iNPH patients, although further studies are needed to clarify this mechanism of VF disturbance.

The mean ICP of the iNPH patients in the present study was relatively low, at 9.0 ± 1.7 mmHg (123 ± 23 mmH_2_O), compared to previous reports, such as that by Berdahl *et al*.; they reported that in POAG and non-glaucomatous patients, the mean ICPs were 9.1 ± 2.9 mmHg (124 ± 39 mm H_2_O) and 13.0 ± 4.2 mmHg (177 ± 57 mm H_2_O), respectively (P < 0.00005)^31^. We therefore cannot exclude the possibility that a lower ICP as an important parameter in the pathophysiology of NTG in iNPH patients. Previous reports have shown rapid progression of NTG in iNPH patients after VP shunt placement, which may result in a rapid decrease of ICP or fluctuations^12^. Although the difference was not significant, the concentration of p-tau was relatively higher in NTG+/iNPH patients compared to NTG-/iNPH patients in this study (Table 2). It seems reasonable to postulate that the presence of iNPH implies an underlying susceptibility to axonal injury in optic nerve tissues, possibly due to decreased CSF turnover and accumulation of neurotoxic substances, with lower ICP or fluctuations therein possibly modifying axonal susceptibility in the optic nerve.

We also found that the mean cupping depth of the optic disc of iNPH−/NTG+ patients was significantly greater than those of iNPH+/NTG+ patients in an age-matched linear combination model (Figs 1 and 3). Generally, in the glaucomatous optic nerve, cupping of the optic disc, and the depth thereof, reflect a loss of RGC axons and a posterior bowing of the lamina cribrosa, accompanied by extensive remodeling of the optic nerve head with disease progression^39^. There are several possible reasons for this significantly shallower cupping depth of the optic disc in iNPH+/NTG+ patients. First, a relatively high CSFP (although still in the normal range) in iNPH+/NTG+ patients, as stated above, may play a role: the cupping depth of the disc is known to be much greater with progression of glaucoma, reflecting optic nerve damage. The TPG reportedly plays an important role in the progression of optic nerve damage, where the more the TPG fluctuates, the more optic nerve damage occurs due to disturbed axonal transportation or metabolism in ganglion cells^40,41^. It has been reported that an 11 mmHg change in the ICP is equal to a 1 mmHg change in the IOP^42^, and the reported ICP in NTG patients was lower than in the present results; this suggests that TPG fluctuations may not play an important role in the generation of NTG compared to iNPH−/NTG+ patients, because of the relatively higher CSFP. Another important factor is the particular condition of the CSF, and the CSF turnover, seen in iNPH patients, where accumulated neurotoxic substances may play a more important role than in iNPH−/NTG+ patients. As stated above, subsequent disturbance of axon flow, or an inflammatory reaction from supportive glial cells, could result in a progressively more shallow cupping depth of the optic disc, as seen in the present study. Still, we should keep in mind that there exists the possibility of iNPH−induced cupping is not actually due to glaucoma.

Collectively, although further study is needed, the pathophysiological profile of iNPH+/NTG+ and iNPH−/NTG+ patients may differ at least in terms of these parameters.

Our study had several limitations. First, we could not measure the ICP in NTG patients. In addition, although we matched the C/D ratio, MD, and age between the iNPH+/NTG and iNPH−/NTG groups, and glaucoma diagnosis was made not only by the VF test but also based on disc morphology and OCT results, the reliability of the VF test was relatively low in iNPH patients due to the nature of iNPH as a dementia-related disease (data not shown). Care must therefore be taken when interpreting the results. Second, this study only included Japanese patients, in which the prevalence of NTG is relatively high, so our results cannot be applied directly to other ethnic groups. In our results, the patients’ ratio for iNPH+/NTG+ is much higher than the other prior studies. As stated above, we made glaucoma diagnosis not only by the VF test but also based on disc morphology and OCT results. As a result, NTG patients with early glaucomatous changes including preperimetric glaucoma, were included. In addition, as stated above, there exists considerable possibility that the mechanisms of iNPH−induced cupping differ from common glaucomatous mechanisms. Therefore, the higher ratio of NTG among iNPH patients in the present study may be reflecting not only the higher prevalence of NTG in Japanese patients but also the methods for glaucoma diagnosis in the present study. Finally, this was a cross-sectional, observational study and we could not follow the patients for long periods. Further case-control studies will be needed to confirm the effect of the tap-test and the prognosis of NTG+/iNPH patients.

In conclusion, we found a significantly higher prevalence of NTG in iNPH patients, and the ICP was higher in the iNPH+/NTG+ group compared to the iNPH+/NTG-group. We also found a significant difference between iNPH−/NTG and iNPH+/NTG+ patients in terms of the cupping depth of the optic disc. Further studies are needed to clarify how decreased CSF turnover and ICP play a pathogenic role in the development of NTG, but the data from iNPH patients may provide important insights to identify the mechanism underlying optic nerve damage in NTG patients.

## Patients and Methods

### Subjects

This prospective observational cross-sectional study was conducted at University of Tokyo Hospital and Kashiwa Tanaka Hospital. The Institutional Review Boards and Ethics Committees of both institutes approved the study, and the protocol adhered to the tenets of the Declaration of Helsinki. Written informed consent was obtained from each participating patient.

All iNPH patients who were diagnosed and treated for iNPH at Kashiwa Tanaka Hospital (Chiba, Japan) from April to June 2016 were serially recruited in this study. Ophthalmological examination was performed as a routine examination prior to the iNPH diagnosis. No iNPH patients had a history of elevated IOPs, glaucoma medication or other treatments, including surgery, and all iNPH patients with NTG were newly diagnosed glaucoma patients. Finally, 20 iNPH patients and 16 age-matched NTG patients without iNPH (iNPH−/NTG+ patients), who were diagnosed at the University of Tokyo Hospital, were serially included in the study (Table 1). Of the 20 iNPH patients, 11 were diagnosed with glaucoma with a normal IOP. We compared 22 glaucomatous eyes (11 patients) with iNPH (iNPH+/NTG+) and 27 age-matched NTG eyes (16 patients) without iNPH (iNPH−/NTG+). All patients were treated between 2016 and 2017 and underwent an ocular examination, including an autorefractometry examination, measurement of best-corrected visual acuity, slit-lamp examination, measurement of the axial length using the IOL Master instrument (Carl Zeiss Meditec, Dublin, CA, USA), measurement of the IOP, fundoscopy, visual field (VF) measurement using the Humphrey Field Analyzer (Carl Zeiss Meditec), and a spectral domain-optical coherence tomography (SD-OCT) examination using an RS-3000 Advance OCT instrument (software version 1.4.2.1; NIDEK, Gamagori, Japan). Diagnoses of glaucoma for both patient groups were made by two glaucoma specialists at the University of Tokyo Hospital, and the diagnosing criteria of NTG were the same for both NTG groups with or without iNPH. Of the POAG patients, who had a glaucomatous visual field or optic disc, as well as a normal angle with gonioscopy, and normal IOP without any cause were diagnosed with NTG. For iNPH patients, all ophthalmic data were collected prior to the beginning of iNPH treatment, to exclude the possibility that iNPH treatment affected the progression of glaucoma^16,22^.

### Diagnosis of iNPH and ICP measurement

The diagnosis of iNPH was made by one specialist in neurology at Kashiwa Tanaka Hospital according to previously published diagnostic criteria^22,43^. All iNPH patients underwent clinical data collection, a CSF tap-test, and a magnetic resonance imaging examination for CSF volume analysis. The CSF tap-test involved the removal of 30–40 mL of CSF via a lumbar tap, and the ICP was measured at the site of puncture. The concentration of phosphorylated tau protein (p-tau) in the collected CSF was also determined. Responses to the CSF tap-test were assessed using a Japanese idiopathic NPH grading scale or the Mini-Mental State Examination (MMSE), and by quantitative examination of gait and cognition before and after the CSF tap-test.

### OCT measurements and assessments of the optic discs

OCT examinations were performed using SD-OCT. Optic nerve appearance was classified according to past reports^44–47^. The cupping depth of the disc was measured according to past reports^48–52^, using SD-OCT as shown in Fig. 4. On the acquired B-scan image, Bruch’s membrane was first marked, and the Bruch’s membrane openings were connected to form a reference plane. The distance from the reference plane to the bottom of the optic disc was taken as the cupping depth of the disc.

### Statistical analysis

Data were statistically analyzed using Easy R software (http://www.jichi.ac.jp/saitama-sct/SaitamaHP.files/statmedEN.html)^53^. To determine the relationship between ICP and the prevalence of NTG, and how iNPH affected the cupping depth of the disc, a linear combination model was used to compare the age-matched iNPH−/NTG+ group with the iNPH+/NTG+ group. For the linear combination model, the cup/disc (C/D) ratio was matched for the iNPH+/NTG+ and iNPH−/NTG+ patients, so that the same glaucoma stage could be used for comparisons. A linear combination model was applied to a nested dataset, and patients were treated as a “random effect” because both eyes were included in the analyses.

The mean values and standard deviations, for age and other characteristics and indices, were calculated for the iNPH and iNPH−/NTG+ groups. These parameters were compared among the iNPH+/NTG-, iNPH+/NTG+, and iNPH−/NTG+ patients using the Mann-Whitney U test, chi-square or Fisher’s exact test, because the majority of the data did not show a normal distribution according to the Shapiro-Wilk test. Differences in the data among the groups were analyzed by one-way analysis of variance (ANOVA), with Tukey’s post-hoc test applied. A value of p < 0.05 was regarded as statistically significant in all analyses.

## References

[1] Quigley HA, Broman AT (2006). The number of people with glaucoma worldwide in 2010 and 2020. Br J Ophthalmol..

[2] Kwon YH, Fingert JH, Kuehn MH, Alward WL (2009). Primary open-angle glaucoma. N Engl J Med..

[3] Weinreb RN, Khaw PT (2004). Primary open-angle glaucoma. Lancet..

[4] Tomita G (2000). The optic nerve head in normal-tension glaucoma. Curr Opin Ophthalmol..

[5] McCulley TJ, Chang JR, Piluek WJ (2015). Intracranial pressure and glaucoma. J Neurophthalmol. 35.

[6] Soldatos T, Chatzimichail K, Papathanasiou M, Gouliamos A (2009). Optic nerve sonography: a new window for the non-invasive evaluation of intracranial pressure in brain injury. Emerg Med J..

[7] Tayal VS (2007). Emergency department sonographic measurement of optic nerve sheath diameter to detect findings of increased intracranial pressure in adult head injury patients. Ann Emerg Med..

[8] Véronique Promelle (2016). Ocular blood flow and cerebrospinal fluid pressure in glaucoma. Acta Radiol Open..

[9] Berdahl JP, Fautsch MP, Stinnett SS, Allingham RR (2008). Intracranial pressure in primary open angle glaucoma, normal tension glaucoma, and ocular hypertension: a case-control study. Invest Ophthalmol Vis Sci..

[10] Ren R (2010). Cerebrospinal fluid pressure in glaucoma: a prospective study. Ophthalmology..

[11] Chen BH, Drucker MD, Louis KM, Richards DW (2016). Progression of Normal-Tension Glaucoma After Ventriculoperitoneal Shunt to Decrease Cerebrospinal Fluid Pressure. J Glaucoma..

[12] Gallina P (2017). Glaucoma in patients with shunt-treated normal pressure hydrocephalus. J Neurosurg..

[13] Lindén C (2018). Normal-Tension Glaucoma Has Normal Intracranial Pressure: A Prospective Study of Intracranial Pressure and Intraocular Pressure in Different Body Positions. Ophthalmology..

[14] Hayreh SS (2009). Cerebrospinal fluid pressure and glaucomatous optic disc cupping. Graefes Arch Clin Exp Ophthalmol..

[15] Wostyn P, De Groot V, Van Dam D, Audenaert K, De Deyn PP (2013). Senescent changes in cerebrospinal fluid circulatory physiology and their role in the pathogenesis of normal-tension glaucoma. Am J Ophthalmol..

[16] Chang TC, Singh K (2009). Glaucomatous disease in patients with normal pressure hydrocephalus. J Glaucoma..

[17] Wostyn P, Audenaert K, De Deyn PP (2010). High occurrence rate of glaucoma among patients with normal pressure hydrocephalus. J Glaucoma..

[18] Preston JE (2001). Ageing choroid plexus-cerebrospinal fluid system. Microsc Res Tech..

[19] Silverberg GD, Mayo M, Saul T, Rubenstein E, McGuire D (2003). Alzheimer’s disease, normal-pressure hydrocephalus, and senescent changes in CSF circulatory physiology: a hypothesis. Lancet Neurol..

[20] Wostyn P, Audenaert K, De Deyn PP (2008). Alzheimer’s disease-related changes in diseases characterized by elevation of intracranial or intraocular pressure. Clin Neurol Neurosurg..

[21] Brown PD, Davies SL, Speake T, Millar ID (2004). Molecular mechanisms of cerebrospinal fluid production. Neuroscience..

[22] Bokhari RF, Baeesa SS (2013). Does the treatment of normal pressure hydrocephalus put the retinal ganglion cells at risk? A brief literature review and novel hypothesis. Med Hypotheses..

[23] Bayer AU, Ferrari F, Erb C (2002). High occurrence rate of glaucoma among patients with Alzheimer’s disease. Eur Neurol..

[24] Tamura H (2006). High frequency of open-angle glaucoma in Japanese patients with Alzheimer’s disease. J Neurol Sci..

[25] Helmer C (2013). Is there a link between open-angle glaucoma and dementia? The Three-City-Alienor cohort. Ann Neurol..

[26] Iwase A (2004). The prevalence of primary open-angle glaucoma in Japanese: the Tajimi Study. Ophthalmology..

[27] Bizrah M, Li G, Cordeiro MF (2011). Glaucoma and Alzheimer’s Disease in the Elderly. Aging Health..

[28] Trenkwalder C (1995). Starnberg trial on epidemiology of Parkinsonism and hypertension in the elderly. Prevalence of Parkinson’s disease and related disorders assessed by a door-to-door survey of inhabitants older than 65 years. Arch Neurol..

[29] Bech-Azeddine R (2001). Idiopathic normal-pressure hydrocephalus: evaluation and findings in a multidisciplinary memory clinic. Eur J Neurol..

[30] Iseki C (2009). Asymptomatic ventriculomegaly with features of idiopathic normal pressure hydrocephalus on MRI (AVIM) in the elderly: a prospective study in a Japanese population. J Neurol Sci..

[31] Berdahl JP, Allingham RR, Johnson DH (2008). Cerebrospinal fluid pressure is decreased in primary open-angle glaucoma. Ophthalmology..

[32] Lenfeldt N, Koskinen LO, Bergenheim AT, Malm J, Eklund A (2007). CSF pressure assessed by lumbar puncture agrees with intracranial pressure. Neurology..

[33] Hannes Stephensen (2005). Objective B wave analysis in 55 patients with non-communicating and communicating hydrocephalus. J Neurol Neurosurg Psychiatry..

[34] Triyoso DH, Good TA (1999). Pulsatile shear stress leads to DNA fragmentation in human SH-SY5Y neuroblastoma cell line. J Physiol..

[35] Asrani S (2000). Large diurnal fluctuations in intraocular pressure are an independent risk factor in patients with glaucoma. J Glaucoma..

[36] Serot JM, Zmudka J, Jouanny P (2012). A possible role for CSF turnover and choroid plexus in the pathogenesis of late onset Alzheimer’s disease. J Alzheimers Dis..

[37] Silverberg GD (2004). Normal pressure hydrocephalus (NPH): ischaemia, CSF stagnation or both. Brain..

[38] Hou R (2016). Intracranial pressure (ICP) and optic nerve subarachnoid space pressure (ONSP) correlation in the optic nerve chamber: the Beijing Intracranial and Intraocular Pressure (iCOP) study. Brain Res..

[39] Morrison JC (2006). Integrins in the optic nerve head: potential roles in glaucomatous optic neuropathy (an American Ophthalmological Society thesis). Trans Am Ophthalmol Soc..

[40] Wostyn P, De Groot V, Audenaert K, De Deyn PP (2011). Are intracranial pressure fluctuations important in glaucoma?. Med Hypotheses..

[41] Lina Siaudvytyte (2014). The Difference in Translaminar Pressure Gradient and Neuroretinal Rim Area in Glaucoma and Healthy Subjects. J Ophthalmol..

[42] Sheeran P, Bland JM, Hall GM (2000). Intraocular pressure changes and alterations in intracranial pressure. Lancet..

[43] Dubois B (2007). Research criteria for the diagnosis of Alzheimer’s disease: revising the NINCDS-ADRDA criteria. Lancet Neurol..

[44] Nicolela MT, Drance SM (1996). Various glaucomatous optic nerve appearances: clinical correlations. Ophthalmology..

[45] Broadway DC, Drance SM, Parfitt CM, Mikelberg FS (1998). The ability of scanning laser ophthalmoscopy to identify various glaucomatous optic disk appearances. Am J Ophthalmol..

[46] Nakazawa T (2010). Different types of optic disc shape in patients with advanced open-angle glaucoma. Jpn J Ophthalmol..

[47] Tanito M (2017). Differentiation of glaucomatous optic discs with different appearances using optic disc topography parameters: The Glaucoma Stereo Analysis Study. PLoS One..

[48] Kim YW, Jeoung JW, Girard MJA (2016). Jean Martial Mari, Ki Ho Park. Positional and Curvature Difference of Lamina Cribrosa According to the Baseline Intraocular Pressure in Primary Open-Angle Glaucoma: A Swept-Source Optical Coherence Tomography (SS-OCT) Study. PLoS One..

[49] Kim YW, Kim DW, Jeoung JW, Kim DM, Park KH (2015). Peripheral lamina cribrosa depth in primary open-angle glaucoma: a swept-source optical coherence tomography study of lamina cribrosa. Eye (Lond)..

[50] Lee EJ, Choi YJ, Kim T-W, Hwang J-M (2016). Comparison of the Deep Optic Nerve Head Structure between Normal-Tension Glaucoma and Nonarteritic Anterior Ischemic Optic Neuropathy. PLoS One..

[51] Naoko Takada (2016). OCT-Based Quantification and Classification of Optic Disc Structure in Glaucoma Patients. PLoS One..

[52] Prata TS (2017). *In vivo* analysis of glaucoma-related features within the optic nerve head using enhanced depth imaging optical coherence tomography. PLoS One..

[53] Kanda Y (2013). Investigation of the freely available easy-to-use software ‘EZR’ for medical statistics. Bone Marrow Transplant..

